# Maternal *Clostridium butyricum* supplementation during late gestation and lactation enhances gut bacterial communities, milk quality, and reduces piglet diarrhea

**DOI:** 10.1016/j.csbj.2025.06.040

**Published:** 2025-06-25

**Authors:** Morakot Nuntapaitoon, Piraya Chatthanathon, Matanee Palasuk, Alisa Wilantho, Jakavat Ruampatana, Sissades Tongsima, Sarn Settachaimongkon, Naraporn Somboonna

**Affiliations:** a Department of Obstetrics, Gynaecology and Reproduction, Faculty of Veterinary Science, Chulalongkorn University, Bangkok, Thailand; b Multi-Omics for Functional Products in Food, Cosmetics and Animals Research Unit, Chulalongkorn University, Bangkok 10330, Thailand; c Department of Microbiology, Faculty of Science, Chulalongkorn University, Bangkok, Thailand; d Microbiome Research Unit for Probiotics in Food and Cosmetics, Chulalongkorn University, Bangkok, Thailand; e National Biobank of Thailand, National Center for Genetic Engineering and Biotechnology (BIOTEC), National Science and Technology Development Agency, Pathum Thani 12120, Thailand; f Department of Food Technology, Faculty of Science, Chulalongkorn University, Bangkok, Thailand; g Omics Sciences and Bioinformatics Center, Faculty of Science, Chulalongkorn University, Bangkok 10330, Thailand

**Keywords:** *Clostridium butyricum*, Microbiota, Gut microbiome, Feces, Sow, Milk, Metabolome

## Abstract

**Experimental objective:**

Diarrhea is a major cause of piglet mortality, often reported associated with maternal gut bacterial communities (microbiota). Maternal supplementation with probiotic *Clostridium butyricum* during late gestation showed to reduce piglet diarrhea during the suckling period. This study thereby investigated the effects of probiotic supplementation on sow gut (feces) microbiota and their potential microbial metabolisms.

**Methods:**

Sow and litter performances, including milk compositions and incidences of piglet diarrhea, were recorded from farrowing to weaning of control- supplemented vs. probiotic-supplemented sows. Fecal samples from sows classified as before (Cb=17) and after (Ca=17) probiotic supplementation were analyzed using 16S rRNA gene sequencing and 16S rRNA qPCR, following bioinformatic analyses for alpha-beta diversity, quantitative microbiota, LEfSe (Linear discriminant analysis Effect Size) taxon biomarker analysis, potential microbial metabolism profiles, and statistical correlations with microbial species and clinical data performances.

**Results:**

Probiotic-supplemented sows demonstrated the greater average piglets born alive and lower mummified fetuses (*P* > 0.05), and the statistical higher protein and casein contents in their colostrum (*P* < 0.05). Following microbiota analyses, no significant difference was observed in operational taxonomic units (OTUs), Chao1, and Shannon alpha-diversity indices between Cb and Ca samples. Nevertheless, Ca sows exhibited higher relative abundances of *Clostridium*, SMB53, g_*Turicibacter*, *Treponema*, *Bacillus*, *Enterococcus* and *Lactobacillus*, while the lower abundances of *Oscillospira*, *Prevotella*, *Phascolarctobacterium* and *Ruminococcus*, compared with Cb sows. This highlighted that after the probiotic supplementation showed the sow gut microbiota more abundances of potentially beneficial bacteria, including the supplemented probiotic *C. butyricum*, g_*Bacillus*, g_*Enterococcus* and g_*Lactobacillus*, for instances. The finding was consistent with the LEfSe (Linear discriminant analysis Effect Size) taxon biomarker analysis for the Ca group. Several microbial related metabolic pathways in sow feces were altered after probiotic supplement, particularly relevant to amino acid and short-chain fatty acid metabolisms (i.e*.,* propanoate and butanoate), xenobiotics biodegradation and lipid metabolism. Supportively, the gut microbiota changes of Ca sows might associate with improved sow performance and milk metabolomic profile.

**Conclusions:**

The maternal probiotic *C. butyricum* supplementation during late gestation and lactation showed the improved sows’ intestine, milk components, and the reduced piglet diarrhea cases. This helps to understand and support the probiotic supplementation in sows.

## Graphical Abstract

**Figure:**
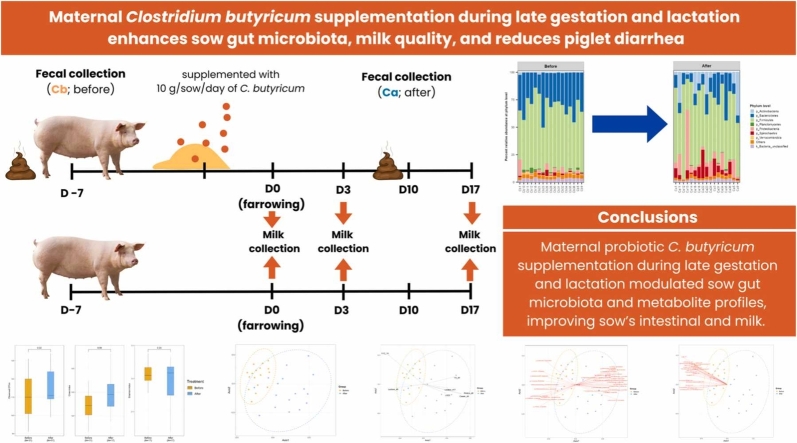

## 1 Introduction

Piglet diarrhea causes a huge economic challenge to swine industry due to increased mortality, weight loss, slow growth, and treatment costs [1]. Stressors, such as environmental changes, dietary shifts, maternal health, and management practices, could negatively impact piglets’ growth performance and health [2]. Maternal health during gestation and lactation is crucial to the development of piglets’ gut health, immunity, and diarrhea incidence. Studies reported unhealthy sow had altered gut and milk bacterial communities (microbiota), which were associated with intestinal dysbiosis and disrupted intestinal barrier in piglets and their susceptibility to enteric infections [2], [3], [4]. Gut microbiota in the intestines play essential roles in nutrient metabolisms, immune modulation, and resistance to pathogens [5], [6]. Dysbiosis of gut microbiota in neonates increase risk of pathogen colonization by *Escherichia coli*, *Clostridium difficile*, rotavirus, coronavirus and *Cryptosporidium* spp., which are major causes of diarrhea [1], [7], [8]. In the last few years, antibiotics have been used in controlling piglet diarrhea; however, antibiotics promotes an emergence of antibiotic-resistant bacteria, which could overgrow the commensal beneficial bacteria in swine industries [9]. Hence, another effective alternative to antibiotic usage to reduce the incidence of piglet diarrhea is important, such as maternal immunization, microbial supplementation to sow or piglet, and farm hygiene [10], [11].

Probiotics, such as *Lactobacillus plantarum, Saccharomyces cerevisiae* and *Clostridium butyrivum*, have been widely used over the past decade to treat piglet diarrhea [12]. Their primary mechanism involves balancing gut microbiota by promoting beneficial bacteria and inhibiting pathogenic bacteria through production of bactericidal and/or bacteriostatic substances (e.g., short-chain fatty acids and bacteriocins), and thus preventing opportunistically pathogenic microbes from colonizing the host intestine [13], [14]. Probiotics, such as *Bacillus licheniformis* and *C. butyricum*, also provide benefits, including enhancing immunity (e.g., microbial and metabolite transfer via milk), improving growth performance in pigs across different ages, and increasing the quality of colostrum and milk [8], [15], [16], [17]. *C. butyricum* is a gram-positive, obligate anaerobic bacteria known for its beneficial effects on gut health, via butyrate production, spore formation, promotion of other gut’s beneficial bacteria, and inhibition of gut’s pathogens [18]. The *C. butyricum*‘s ability to form endospores allow the bacteria to resist extreme conditions, such as low pH, high bile concentrations, and co-administered antibiotics [19]. Studies suggested that *C. butyricum* endospore can rapidly germinate and become metabolically active for functions in the gut environment, enabling it to resume, for examples, butyrate production and exert inhibitory effects on pathogens [19], [20]. This supported their use as one of the swine probiotic supplements. As a feed additive, *C. butyricum* has been shown to enhance growth performance, modulate intestinal microbiota, and improve colostrum and milk metabolite profiles, ultimately reducing diarrhea incidence in pigs [4], [16], [19], [21]. One key microbial mechanism supporting *C. butyricum* supplement to reduce pig’s diarrhea, is its ability to produce high levels of butyrate, which further strengthens the intestinal barrier function by reducing gut permeability [4]. In our previous study highlighted that the piglets nursed by sows supplemented with *C. butyricum* exhibited the lower diarrhea scores compared to those nursed by non-supplemented sows [16]. Nonetheless, the mechanisms underlying sows’ gut microbiota and their alterations that might associate with these clinical improvements remain unclear. Late gestation and lactation represent critical periods for piglet development, as this is when maternal microbial and metabolite transfer through the placenta, birth canal, and milk, provided that milk presents one influence on piglet gut colonization and immune system maturation [16], [17]. The present study therefore analyzed the effects of probiotic *C. butyricum* supplementation in late-gestating sows’ intestines (feces) on the bacterial diversity and compositions (sows’ gut microbiota), along the sow’s gut quantitative microbiota, the sow fecal metabolism, as well as their potential correlation with clinical data on the sow reproductive performance and milk metabolomic profiles. This study helps strengthen if probiotic supplementation during the late gestation and lactation periods provide a strategic time point to modulate maternal gut environments for reproductive performance and piglet health outcome.

## 2 Materials and methods

### 2.1 Animal experiment

**Fig. 1:**
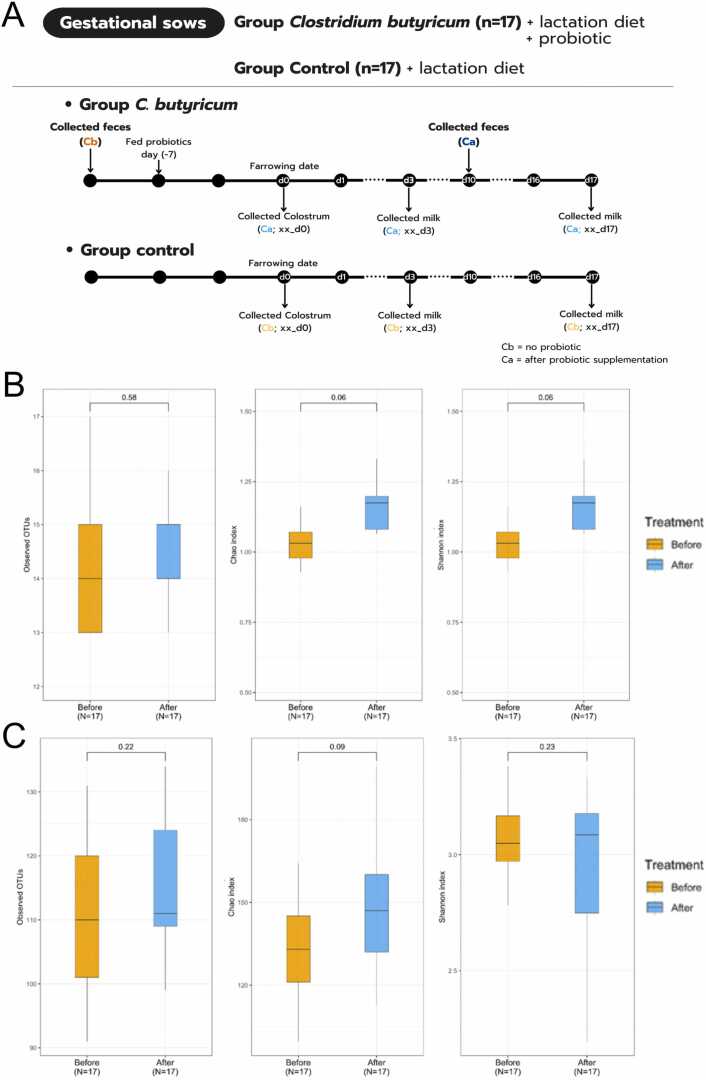
Schematic diagram of experimental groups and timelines (**A**), and alpha diversity estimates of sows’ gut microbiota at phylum (**B**) and genus (**C**) levels, of Cb (n = 17) and Ca (n = 17) gut (feces) microbiota. Alpha diversity estimates included observed OTUs, Chao1, and Shannon indices, and were expressed in boxplot formats where the lines in boxes represent medians. Statistical significance was calculated by student’s *t*-test (*P* < 0.05).

This study was conducted on a commercial swine farm with conventional evaporative cooling system (housing temperature averagely around 28.8°C) in western Thailand. The experimental protocol was approved by the National Research Council of Thailand (Approval number 2031056). Thirty-four gestating sows (Landrace × Yorkshire crossbred) were included, and were allocated to two groups comprising control-supplemented sows (n = 17; sows were fed a standard lactation diet without probiotics), and probiotic-supplemented sows (n = 17; sows fed the same diet as the control group, and supplemented with probiotic *C. butyricum* strain MIYAIRI 588 (FERM BP-2789) isolated from soil in Japan (TopGut®, Huvepharma, Bangkok, Thailand) by top dressing at 10 g/day (3.2 × 109 CFU/g) from 7 days before farrowing date to weaning (29.4 ± 4.5 days)) (see Infographic sketch, and Fig. 1**A**) [16]. Throughout this study, no feed refusals were observed in probiotic-supplemented sows. Sows were housed in individual stalls (1.50 m^2^) during gestation as established protocols [16], [17].

### 2.2 Fecal sample collections

A total of 34 fecal samples were collected from probiotic-supplemented sows on the day before supplementation (Cb, 17 samples) and after probiotic-supplementation for 17 days (Ca, 17 samples). All fecal samples were collected via rectal method from the sows each in 5 mL centrifuge tube, using aseptic techniques. The samples were immediately placed at 4 °C and sent to the laboratory within 6 h for further analyses. Feces were stored at −80 °C for further analysis.

### 2.3 Colostrum and milk sample collections

Colostrum was manually collected from each functional teat (15 mL) within 1 h after the onset of parturition. Milk samples (15 mL each) from all functional teats were collected on days 3 and 17. The udders were cleaned with 75 % alcohol before sample collections. Additionally, sows were administered an intravenous injection of 0.2 mL of oxytocin (10 IU/mL, VetOne, Idaho, USA) at the median auricular vein in the ear before milk sample collection. A total of 102 colostrum and milk samples were collected (51 samples from control-supplemented sows and 51 samples from probiotic-supplemented sows; Fig. 1**A**), and each sample was filtered through gauze to remove particles and debris and stored in a sterile bottle at −20 ℃ until later analysis. The colostrum and milk compositions at days 0, 3, and 17 of lactation was measured fat, proteins, lactose, total solid (TS), and casein (% wt/wt) using infrared spectroscopy (MilkoScan, Foss Electric, Hillerød, Denmark). Noted that fat, protein, lactose and casein are major components of sow colostrum and milk; particularly protein and casein could reflect the amino acid profile and amounts of bioactive peptides or metabolites that influence intestinal health in piglets [16], [23]. Moreover, the colostrum and milk metabolomic profile at days 0, 3, and 17 was analyzed by ^1^H-NMR spectroscopy (Bruker, Rheinstetten, Germany), and all metabolites were measured and published previously [16].

### 2.4 Piglet’s fecal consistency

The fecal consistency was assessed following the method described by Cappai et al. [22] and scored on a scale: 0 = solid, 1 = semi-solid, 2 = semi-liquid, and 3 = liquid. Piglets with a fecal consistency score of ≥ 2 were classified as having diarrhea, while scores 0 and 1 were considered normal fecal. The proportion of piglet diarrhea was calculated using the adapted protocol by Ruampatana et al. [23], as in Eq. (1):

(1) $$\text{Piglet diarrhea proportion }(\%)\;=\;\frac{\text{Number of piglets with diarrhea in each pen}}{\text{Litter size}}\;\times 100$$

### 2.5 Metagenomic DNA extraction and quality examination

Metagenomic DNA was extracted from each fecal sample (0.25 g), using DNeasy PowerSoil Kit, following the manufacturer's instructions (Qiagen, Maryland, USA). The DNA concentration and purification were examined by 1 % (w/v) agarose gel electrophoresis and nanodrop spectrophotometry at A260 and A260/ A280, respectively. The metagenomic DNA were stored at −20 ᵒC [24].

### 2.6 Quantitative count of total bacteria

For a quantitative count of the total bacteria in copy unit, the 16S rRNA gene quantitative polymerase chain reaction (qPCR) was performed using universal primers 1392 F (5′-GYACACACCGCCCGT-3′) and 1492 R (5′-GGTTACCTTGTTACGACTT-3′) [24], [25]. The qPCR thermocycling conditions were 95 °C 5 min, followed by 40 cycles of 95 °C 30 s, 55 °C 45 s, and 72 °C 45 s, and ended with a melting curve analysis to validate a single proper amplicon peak (i.e., neither primer-dimer nor non-specific amplification). The standard quantification curves were generated based on *E. coli* to calculate total bacterial counts. Briefly, the approximately 100-base pair (bp) 1392F-1492R amplicon fragments of *E. coli* were cloned into pGEM-T-Easy Vector (Promega, Wisconsin, USA), and the recombinant plasmids were transformed into *E. coli* DH5α for expression. The inserted fragments were verified by colony PCR using the primers M13F (on vector) and 1492 R (inserted fragment). Ten-fold serial dilutions of the extracted *E. coli* plasmids (concentrations of 10^3^-10^8^ copies/μL) were used as the references for the bacterial copy number computation based on Eq. (2):

(2) $$\text{Copy number per μL }=\;\frac{\text{concentration }(\text{ng}/\text{μL})\;{\times \;6.023\times 10}^{23}\left(\text{copies}/\text{mol}\right)}{\text{length }(\text{bp})\;\times \;6.6\times 10^{11}(\text{ng}/\text{mol})}$$

The qPCR experiments were performed using Rotor-GeneQ (Qiagen, Hilden, Germany). Three replicates were conducted per reaction. The bacteria copy number of each sample was quantified against the reference standard curve by Rotor-Gene Q Series Software (Qiagen, Hilden, Germany) [24], [25].

### 2.7 16S rRNA gene V3-V4 library preparation and MiSeq sequencing

The 16S rRNA gene sequencing using next-generation sequencing (NGS) was performed to investigate the sows’ gut microbiota compositions in Cb and Ca sows. The 16S rRNA gene hypervariable regions (V3-V4) were amplified with universal prokaryotic primers 341 F

(TCGTCGGCAGCGTCAGATGTGTATAAGAGACAGCCTACGGGNGGCWGCAG) and 805 R (GTCTCGTGGGCTCGGAGATGTGTATAAGAGACAGGACTACHVGGGTATCTAATCC) [24], [25]. Libraries were prepared following the 16S Metagenomic Sequencing Library Preparation Protocol (Illumina, San Diego, CA, USA). Each sample was quantified with Qubit2.0 Fluorometer (Invitrogen, Carlsbad, CA, USA) and pooled together in equimolar proportion for 2 × 250 paired end sequencing using MiSeq Reagent Kits v2 (Illumina, San Diego, USA) and MiSeq system (Illumina, San Diego, USA) at the Omics Sciences and Bioinformatics Center, Faculty of Science, Chulalongkorn university. The 16S rRNA gene sequences in this study were deposited in the NCBI open access Sequence Read Archive database (accession number PRJNA1209561).

### 2.8 Bioinformatic analyses for microbiota, quantitative microbiota, alpha and beta diversity, and potential metabolisms

The forward and reverse paired-end reads were merged into single reads, following Mothur’s Standard Operating Procedure for Miseq [26], [27]. After joining, reads were filtered for quality based on removal of (1) short read lengths of ≤ 100 nucleotides (nt) excluding primer and barcode sequences, (2) ambiguous bases ≥ 8, (3) chimera sequences, and (4) homopolymer of ≥ 8 nt [24], [26]. The quality sequences were aligned against the 16S rRNA gene databases SILVA version 132 to remove sequences of mitochondria, chloroplast and eukaryotic lineages, and Greengenes version 13.8 for taxonomy annotation. Samples were normalized to an equal sequencing depth (13,170 quality sequences per sample).

The gut microbiota compositions were analyzed for alpha diversity and beta diversity, using Mothur version 1.39.1 [26], [27], [28]. The alpha diversity for species richness (Chao1, and number of observed OTUs) and species richness and evenness estimators (Shannon, percent relative abundances, and abundance in copy number), at phylum and genus levels, were analyzed. The count of total bacteria in copy number was analyzed along with the percent microbiota composition to yield the quantitative microbiota (the bacterial copy number of each individual OTU) [24], [25], [28]. The statistically significant differences among the top 15 abundant genera were compared between Cb and Ca sows by Student’s *t*-test (*P* < 0.05). At species level where genus *Clostridium* was Mothur v.1.40.5-identified as unclassified, each respective OTU sequence was further classified species by BLASTN against GenBank non-redundant database. For beta diversity analyses, two-dimension non-metric multidimensional scaling (NMDS) was computed using distance dissimilarity index Bray-Curtis. The NMDS along the representing species and the clinical parameter correlations (including potential guts’ microbial metabolism profiles, and clinical performance data), and linear discriminant analysis effect size (LEfSe) for microbial biomarker identification with pairwise Kruskal–Wallis and Wilcoxon tests, were analyzed following established protocols [26], [28], [29]. Estimates of the microbial metabolism profiles were determined by PICRUSt (Phylogenetic Investigation of Communities by Reconstruction of Unobserved States) based on the reference genome annotations in KEGG (Kyoto Encyclopedia of Genes and Genomes pathways) [30] and statistically compared by STAMP (Statistical Analysis of Metagenomic Profiles) with two-sided Welch’s *t*-test and Storey’s FDR correction [31].

### 2.9 Statistical analysis

Sow reproductive performance, litter, piglet diarrhea, and colostrum and milk compositions were analyzed for statistics using SAS version 9.4 (Cary, NC, USA). The chi-squared test was applied to assess the effect of the probiotic-supplemented sows on piglet fecal consistency on days 0, 3, and 17 of age. For microbiota and quantitative microbiota statistical analyses, a comparison between two groups was performed by Student’s *t*-test, and comparisons among multiple parameters were performed by AMOVA (*P* < 0.05) [25] unless stated.

## 3 Results

### 3.1 Clinical sows’ reproductive performance data and diarrhea scores in piglets

The control-supplemented and probiotic-supplemented sows showed no statistically significant differences in reproductive performance data, although probiotic-supplemented sows had the greater average number of piglets born alive and the lower mummified fetuses per litter (Table 1: *P* > 0.05). The colostrum from the probiotic-supplemented sows showed the statistical higher in protein (*P* = 0.003) and casein (*P* < 0.001), and also their milk showed the mean higher trend in protein and casein but not at statistical difference (Table 2).

**Table 1:** Reproductive performance data of control-supplemented sows and probiotic-supplemented sows (data shown as least-square means ± SEM).

**Parameters**	**Control**	**Probiotic**	***P*****-value**
Number of sows (n)	17	17	
Parity^a^	2.5 ± 1.5	2.6 ± 2.0	
Backfat thickness (mm)			
On day 109 of gestation	21.8 ± 1.5	20.6 ± 1.5	0.575
On day 21 of lactation	17.4 ± 0.9	18.6 ± 0.9	0.329
Total number of piglets born per litter	12.9 ± 0.6	12.9 ± 0.6	0.948
Number of piglets born alive per litter	10.9 ± 0.7	11.5 ± 0.7	0.583
Number of stillborn piglets per litter	0.9 ± 0.4	1.4 ± 0.4	0.441
Number of mummified fetuses per litter	1.0 ± 0.4	0.1 ± 0.4	0.104
Milk yield between day 3–10 of lactation (kg/day)	7.8 ± 0.5	7.6 ± 0.5	0.692
Milk yield between day 10–17 of lactation (kg/day)	7.1 ± 0.4	7.5 ± 0.4	0.556
Number of litters (n)	17	17	
On day 3 of age			
Litter size (piglets/litter)	10.4 ± 0.7	11.4 ± 0.7	0.352
Litter weight (kg)	19.4 ± 1.5	19.3 ± 1.5	0.993
On day 10 of age			
Litter size (piglets/litter)	10.1 ± 0.8	9.0 ± 0.8	0.328
Litter weight (kg)	26.7 ± 2.7	24.6 ± 2.7	0.600
On day 17 of age			
Litter size (piglets/litter)	8.9 ± 0.7	8.2 ± 0.7	0.508
Litter weight (kg)	29.2 ± 3.0	28.3 ± 2.9	0.847
On day 21 of age			
Litter size (piglets/litter)	8.6 ± 0.7	8.2 ± 0.7	0.692
Litter weight (kg)	34.8 ± 3.5	35.8 ± 3.5	0.843
At weaning			
Litter size (piglets/litter)	7.5 ± 0.7	7.6 ± 0.7	0.902
Litter weight (kg)	48.6 ± 4.4	49.0 ± 4.4	0.948

a Parity is presented as a mean ± SD instead of least-square means ± SEM.

**Table 2:** Colostrum and milk composition data of control-supplemented sows and probiotic-supplemented sows (data shown as least-square means ± SEM).

**Parameters**	**Control**	**Probiotic**	***P*****-value**
Day 0 (g/100 g)			
Immunoglobulin G (mg/mL)	39.4 ± 3.6	41.6 ± 3.6	0.664
Immunoglobulin A (mg/mL)	9.7 ± 1.7	10.9 ± 1.7	0.645
Fat	6.1 ± 0.6	5.4 ± 1.0	0.507
Protein	15.8 ± 0.4	18.0 ± 0.6	0.003
Casein	12.5 ± 0.3	14.6 ± 0.4	< 0.001
Lactose	2.4 ± 0.1	2.2 ± 0.2	0.459
Total solid	25.1 ± 0.8	26.1 ± 1.2	0.494
Day 3 (g/100 g)			
Fat	10.6 ± 0.6	11.4 ± 0.8	0.405
Protein	5.7 ± 0.4	6.7 ± 0.4	0.078
Casein	4.1 ± 0.2	4.6 ± 0.3	0.212
Lactose	4.3 ± 0.1	4.3 ± 0.1	0.898
Total solid	22.1 ± 0.8	23.7 ± 0.9	0.179
Day 17 (g/100 g)			
Fat	7.8 ± 0.6	7.2 ± 0.8	0.522
Protein	5.2 ± 0.4	5.1 ± 0.4	0.821
Casein	4.1 ± 0.2	4.2 ± 0.3	0.795
Lactose	4.5 ± 0.1	4.8 ± 0.1	0.157
Total solid	19.3 ± 0.8	18.7 ± 0.9	0.622

**Fig. 2:**
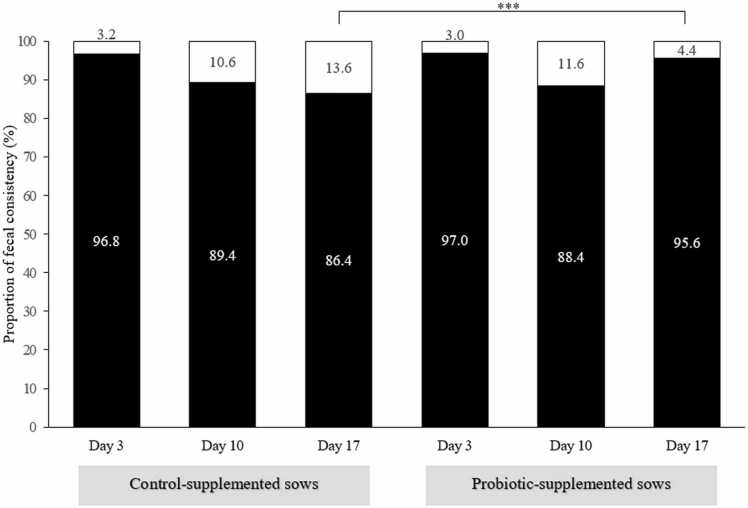
Piglets’ fecal consistency (normal = black; diarrhea = white) on days 3, 10, and 17 of age from comparing control-supplemented and probiotic-supplemented sows. *** represents level of significant differences at *P* < 0.001.

The effect of probiotic-supplemented sows on piglet fecal consistency aged 3, 10 and 17 days exhibited that no statistical difference in piglets’ diarrhea on aged 3 and 10 days from control-supplemented vs. probiotic-supplemented sows (*P* > 0.05). However, the statistically lower diarrhea proportion in piglets were observed on aged 10 days (Fig. 2: 13.6 diarrhea piglets from control-supplemented sows vs. 13.6 diarrhea piglets from probiotic-supplemented sows, *P* < 0.001) [16], [17].

### 3.2 Sequencing results and alpha diversity estimates

The 16S rRNA gene sequencing results of Cb and Ca fecal samples showed 768,891 total quality reads (after Mothur’s SOP, see Materials and Methods for details), with average raw 24,874 reads/sample and average quality 22,164 reads/sample (Table 3). The alpha diversity of gut microbiota based on numbers observed OTUs and Chao1 (OTU richness), at phylum and genus levels, suggested a trend to increase in taxa number after the probiotic supplement (Fig. 1**C**: at genus level, averagely 109.71 taxa in Cb and 114.76 taxa in Ca). Analyzing along with the OTU evenness via Shannon index at phylum and genus levels, Ca showed a similar trend but at the smaller increase difference than the Chao1 (Fig. 1**B** and 1**C**).

**Table 3:** Sample IDs and number of raw and quality sequencing reads of probiotic-supplemented sows before and after probiotic treatment.

**Sample IDs**	**Group**	**Treat**	**Raw reads**	**Quality reads**
Ca01	*Clostridium*	After	18,475	16,982
Ca03	*Clostridium*	After	25,657	23,591
Ca05	*Clostridium*	After	17,812	16,190
Ca09	*Clostridium*	After	20,928	18,950
Ca11	*Clostridium*	After	14,576	13,170
Ca12	*Clostridium*	After	15,975	14,520
Ca13	*Clostridium*	After	22,935	21,254
Ca14	*Clostridium*	After	15,726	14,142
Ca16	*Clostridium*	After	30,725	27,797
Ca18	*Clostridium*	After	23,366	21,112
Ca22	*Clostridium*	After	16,912	15,358
Ca23	*Clostridium*	After	31,275	28,290
Ca24	*Clostridium*	After	22,427	19,782
Ca31	*Clostridium*	After	27,882	25,184
Ca34	*Clostridium*	After	32,075	27,420
Ca35	*Clostridium*	After	30,856	27,834
Ca36	*Clostridium*	After	28,308	25,285
Cb01	*Clostridium*	Before	24,977	23,039
Cb05	*Clostridium*	Before	19,176	17,735
Cb09	*Clostridium*	Before	39,970	36,653
Cb10	*Clostridium*	Before	18,584	17,001
Cb11	*Clostridium*	Before	28,636	26,117
Cb12	*Clostridium*	Before	36,224	33,422
Cb16	*Clostridium*	Before	31,546	28,317
Cb21	*Clostridium*	Before	33,062	30,200
Cb23	*Clostridium*	Before	28,795	26,696
Cb26	*Clostridium*	Before	23,746	21,567
Cb28	*Clostridium*	Before	20,306	18,656
Cb30	*Clostridium*	Before	19,514	17,853
Cb31	*Clostridium*	Before	24,342	22,408
Cb32	*Clostridium*	Before	23,054	21,155
Cb34	*Clostridium*	Before	21,669	19,742
Cb35	*Clostridium*	Before	33,833	30,929
Cb36	*Clostridium*	Before	22,385	20,540
**average**			24,874	22,614
**total**			845,729	768,891
**lowest**			14,576	13,170
**highest**			39,970	36,653

### 3.3 Bacterial composition differences in sows’ gut microbiota at phylum and genus levels

**Fig. 3:**
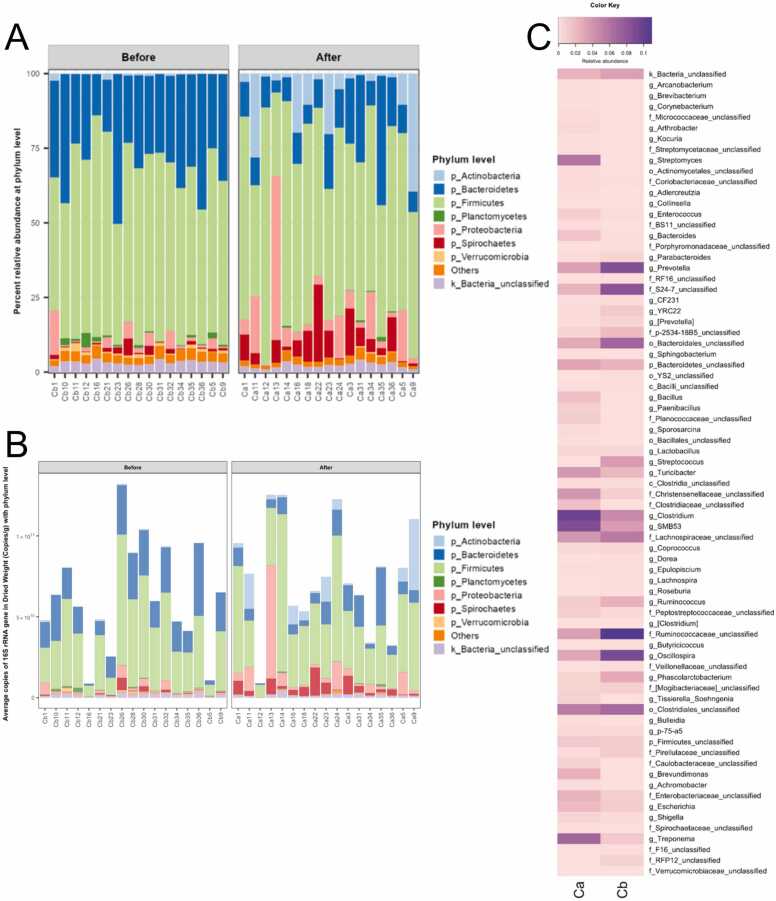
Sows’ gut bacterial compositions in relative percentages (microbiota) and copy numbers (quantitative microbiota) at phylum (**A** and **B**) and genus (**C**) levels. Bacterial phyla that represent less than 1 % of total bacteria, were in “Others”. (**C**), heatmap profiles of bacterial genera in relative abundance (0.001–1.00 scale, alternatively equal to 0.1–100 %) between average Cb and Ca groups, noted that genera with < 0.1 % were not displayed. At genus level, OTUs were classified to the deepest taxonomic level where allowed. k_ abbreviates kingdom; p_, phylum; c_, class; o_, order; f_, family; and g_, genus.

Thirty-two total bacterial phyla were identified; and the relative abundance in percentages and in quantitative copy numbers (quantitative microbiota) were described in Fig. 3**A** and 3**B**, respectively. The Ca demonstrated the relatively higher proportions of phyla Actinobacteria, Firmicutes, Proteobacteria and Spirochetes, and the lower proportion of Bacteroidetes, Planctomycetes and Verrucomicrobia, than the Cb sows (Fig. 3**A**). Quantitative microbiota showed that the average bacterial copy numbers between Cb and Ca remained relatively similar, with the numbers varying across individual sows’ feces. However, the Ca were noticed the consistently small higher trend in total bacterial copy numbers than the Cb, and the bacterial phylum differences remained denoted in the Ca (e.g., increased Actinobacteria (*P* = 0.0153, Proteobacteria (*P* = 0.0949) and Spirochetes (*P* = 0.005); and reduced Bacteroidetes (*P* = 0.0077)) (Fig. 3**B**).

Seventy-five bacterial genera with relative > 0.1 percent abundances were found; and the Ca exhibited 43 genera with increased relative proportions (such as *Streptomyces*, *Bacillus, Lactobacillus*, *Turicibacter*, and *Clostridium*) while 32 genera were decreased (such as *Prevotella*, genera in order Bacteroidales, *Streptococcus*, *Ruminococcus*, and *Oscillospira*), compared with Cb (Fig. 3**C**). Following, the top 15 dominant genera were compared, in percents and copy numbers; and the Ca exhibited the statistically greater genera *Clostridium* (*P* < 0.05), g_SMB53 (was defined the bacteria that belongs Clostridiaceae family, is closely related to *Clostridium*, and is enriched in pigs’ small intestines involved in nutrient metabolisms and short-chain fatty acids (SCFA) production [32] and *Treponema*, in both relative percentage and copy numbers (*P* < 0.05). Conversely, the Ca showed many statistically lower genera, e.g., *Prevotella*, *Oscillospira*, *Phascolarctobacterium* and *Ruminococcus* (*P* < 0.05) (Fig. S1). (Fig. 3**B**).

### 3.4 Beta diversity estimates and representing bacteria taxa analyses

**Fig. 4:**
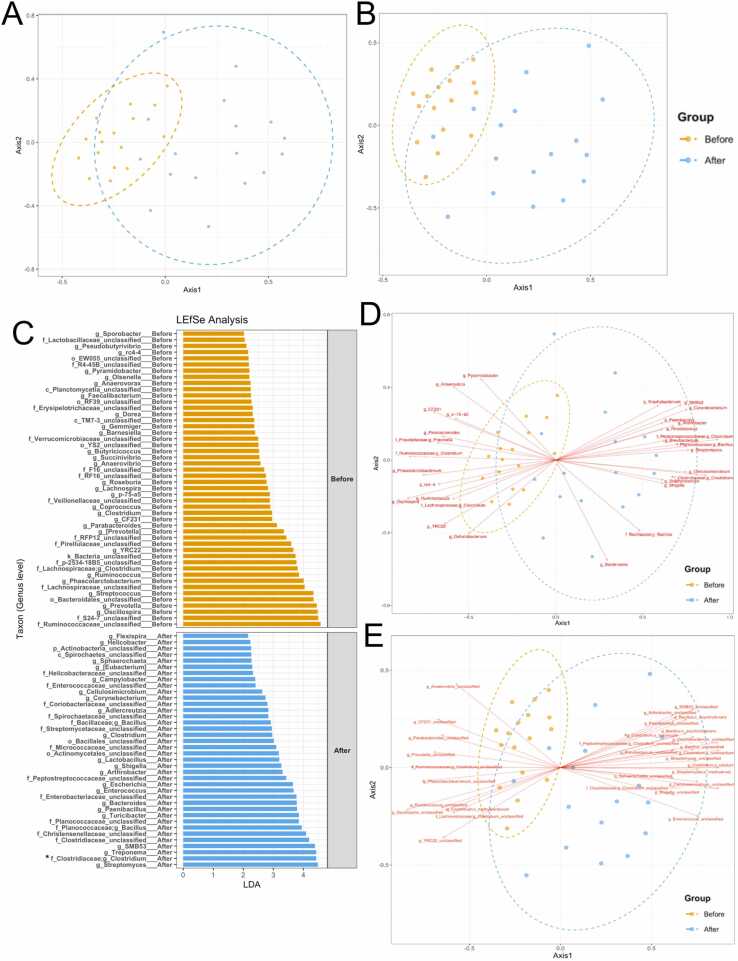
Beta diversity estimates via NMDS at genus (**A**) and species (**B**) levels, genus biomarker analysis LEfSe (**C**), and the statistically associated genus (**D**) and species (**E**) for the Cb and Ca gut microbiota structures (AMOVA, *P* ≤ 0.05). In (**A**, **B**, **D** and **E**), orange and blue circles were automatically drawn at 95 % confidence for Cb and Ca groups, respectively. The direction and length of red arrows in **D** and **E**, indicate the direction and magnitude of the correlation of that bacterial genus (or species) to the Cb (or Ca). For genus (or species) classification, OTUs were classified to the deepest taxonomic level where allowed. k_ abbreviates kingdom; p_, phylum; c_, class; o_, order; f_, family; g_, genus; and s_, species. * on the annotated OTU refers to the BLASTN-confirmed *Clostridium butyricum.*

The non-metric multidimensional scale analyses at genus and species levels demonstrated the rather separated gut microbiota (bacterial community) structures between Cb and Ca, provided that the sows’ gut bacterial diversity after the probiotic supplementation was relatively more diverse and separate to another direction (Fig. 4**A** and 4**B**: *P* > 0.05). Here, the Cb vs. Ca microbiota structures were associated with the predicted LEfSe biomarker for Cb vs. Ca sows. For instances, *Streptococcus*, *Clostridium* (*BLASTN-identified as *C. butyricum*), *Treponema*, *SMB53* and *Lactobacillus* were identified with relatively high linear discriminant analysis score (LDA) > 3.0–4.0 (Fig. 4**C**). Consistently, these species were statistically denoted by Mothur as representing species along the Ca microbiota structure (Fig. 4**E**). On the other hand, other bacterial genera were associated with the Cb microbiota structure, for examples, species in family Ruminococcaceae and species in genera *Oscillospira*, *Prevotella* and *Streptococcus* (Fig. 4**C-**4**E**). Noted that these indicating genera (or species) were consistent with the previous Fig. 3**C** and **S1** results, including *SMB53*, *Streptomyces*, f_Peptostreptococcaceae, *Clostridium* and *Bacillus* that were positively correlated with the Ca, and genera *Oscillospira*, *Prevotella* and *Ruminococcus* that were negatively correlated (meaning correlated with the Cb sows).

### 3.5 Differential Cb and Ca gut microbiota structures associated with potential microbial metabolic functions, sow reproductive performances, and milk quality profiles

**Fig. 5:**
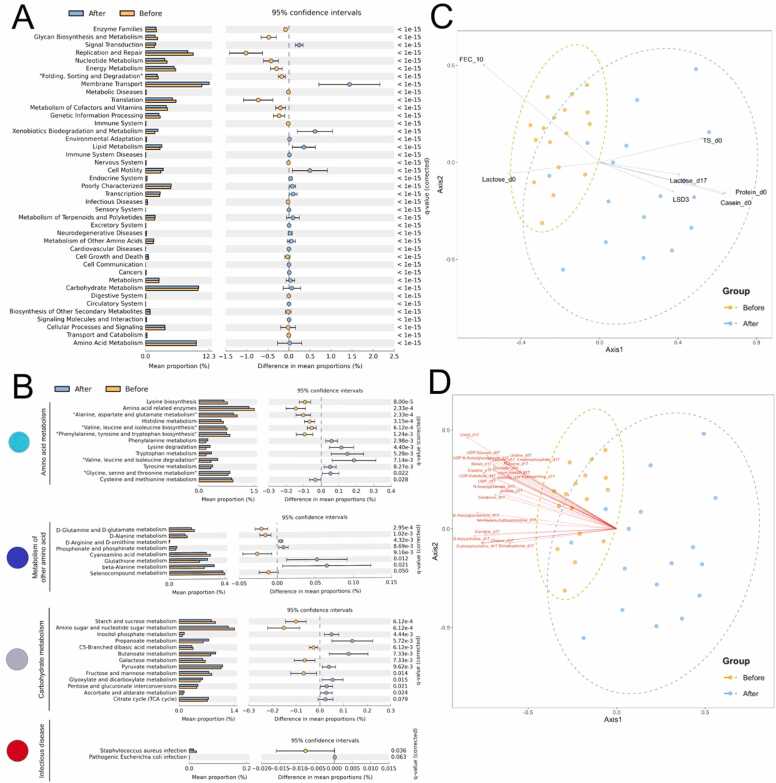
Differential Cb and Ca gut microbiota structures associated with microbial metabolic potentials (**A** and **B**), sow performances (**C**), and milk metabolome profiles (**D**). Microbial metabolic potentials were classified based on KEGG pathway at the 2nd (**A**) and 3rd (**B**) levels; the statistical test was computed by two-sided Welch’s *t*-test with Storey’s FDR correction (*P* ≤ 0.05 in **A** and *P* ≤ 0.079 in **B** were displayed); and the statistical microbial metabolic pathways that were not found as pig diseases were excluded (Tuberculosis, Amebiasis, Epithelial cell signaling in *Helicobacter pylori* infection, Influenza A, and Shigellosis). (**C** and **D**), the NMDS at species level along the Pearson's correlation with pig performances and milk components (Table S2) (*P* ≤ 0.05), and milk metabolome profiles (Table S3) (top 25 milk metabolites, *P* ≤ 0.05). Arrows indicate the direction and magnitude of correlation of each factor to the Ca (or Cb) gut microbiota.

Following the probiotic supplementation, 23 KEGG categories were significantly higher (e.g., membrane transport, xenobiotics biodegradation and metabolism, lipid metabolism, metabolism, carbohydrate metabolism, and amino acid metabolism) and 18 categories were significantly lower (Fig. 5**A**). Indeed, several amino acid and carbohydrate metabolisms were statistically higher in the Ca. Examples of the greater amino acid metabolisms in Ca were: valine, leucine and isoleucine degradation; tryptophan metabolism; lysine degradation; phenylalanine metabolism; tyrosine metabolism; glycine, serine and threonine metabolism; beta-alanine metabolism; and glutathione metabolism; and examples of the greater carbohydrate metabolisms in Ca were: propanoate metabolism, and pyruvate metabolism (Fig. 5**B,** Fig. S2 and Table S1). Infectious microbes that could be related to diarrhea, such as *Staphylococcus aureus*, showed statistically lower in the Ca (*P* < 0.05) (Fig. 5**B**). Fig. S2 showed the *S. aureus* infection clustered with the Cb sows. Moreover, for category of cell communications in eukaryotic process, the microbial metabolic functions involved functions of tight junction (*P* = 0.056), and adherens junction (*P* = 0.056) were positively correlated with the Ca (Figs. 5**A,** 5**B,** and Fig. S2).

The NMDS Pearson’s correlations with sow reproductive performances and milk components indicated a litter size on day 3 (abbreviated LSD3, *P* = 0.031), and milk components of total solid day 0 (TS_d0, *P* = 0.010), lactose day 17 (lactose_d17, *P* = 0.029), protein day 0 (protein_d0, *P* < 0.001) and casein day 0 (casein _d0, *P* = 0.001) as positively correlated with the gut microbiota of the Ca; whereas exhibiting the inverse correlation to feces score on day 10 (FEC_10, *P* = 0.002) and lactose day 0 (lactose_d0, *P* = 0.025) (Fig. 5**C** and Table S2). The sows’ milk metabolome profiles days 0, 3 and 17 of the probiotic-supplemented vs. control-supplemented sows were analyzed for potential Pearson’s correlation with the Cb and Ca gut microbiota structures. The top 25 significant milk metabolites were metabolites at the farrowing day 17, and all inversely correlated with the Ca gut microbiota. These included uracil (*P* = 0.001), UDP-glucose (*P* = 0.010), O-acetyl choline (*P* = 0.003), UDP-N-acetyl glucosamine (*P* = 0.0112), UDP-galactose (*P* = 0.007), N-acetyl glucosamine (*P* = 0.004), sn-glycero-3-phosphocoline (*P* = 0.004), creatine (*P* = 0.011), betain (*P* = 0.016), choline (*P* = 0.006), O-phosphocholine (*P* = 0.006), glycolate (*P* = 0.016), carnitine (*P* = 0.008), uridine monophosphate (UMP) (*P* = 0.016), N-acetyl glutamate (*P* = 0.015), myo-inositol (*P* = 0.023), creatine phosphate (*P* = 0.035), glycine (*P* = 0.035), lactose (*P* = 0.035), uridine (*P* = 0.035), creatinine (*P* = 0.019), acetate (*P* = 0.026), adenine (*P* = 0.043), hypoxanthine (*P* = 0.043), and di-methyl amine (*P* = 0.047) (Fig. 5**D** and Table S3).

## 4 Discussion

Piglet diarrhea is a significant challenge in the swine industry, causing high pre-weaning mortality. During the suckling period, the piglets’ immature immune system and unstable gut microbiota make them susceptible to pathogens and diarrhea [33]. Although dietary antibiotics effectively reduce diarrhea, their overuse has caused drug-resistant bacteria, antibiotic residues, and microbiota imbalances in piglets [6], [8]. As a sustainable alternative, probiotics are gaining attention for mitigating diseases in piglets and pigs by restoring gut microbiota balance and promoting intestinal homeostasis [8], [34].

*C. butyricum,* a gram-positive, rod-shaped, obligate anaerobic bacterium found in the intestines of healthy humans and animals, is widely used as a probiotic to modulate gut microbiota and treat intestinal disorders [35]. In this study, piglets nursed by probiotic-supplemented sows exhibited the lower proportion of diarrhea than those nursed by non-supplemented sows, particularly on day 17 of age. Similarly, our previous study demonstrated that probiotic-supplemented sows had altered colostrum and milk profiles, and reduced piglet diarrhea [16]. Yu et al. [36] studied the different probiotic (*B. licheniformis*) and found that probiotic-supplemented sows during gestation had altered gut microbiota and reduced sows’ postpartum dysgalactia syndrome, which could affect milk production and quality.

The sows’ gut microbiota, quantitative microbiota, as well as their potential microbial metabolisms, were analyzed before and after probiotic supplementation. Our results showed a tendency to increase in the alpha diversity (OTUs, Chao1, and Shannon) although no significant differences, for the Ca. Gut microbiota analyses at the phylum level revealed dominant bacterial phyla, including *Actinobacteria*, *Bacteroidetes*, *Firmicutes*, *Planctomycetes*, *Proteobacteria*, and *Spirochaetes*, consistent with previous studies [33], [37]. Variations in certain phyla, including the core phyla found in this study such as Firmicutes, Bacteroidetes, Proteobacteria and Actinobacteria, were perhaps attributed to age, physiological changes, and geographical factors [38], [39]. For examples, the microbial mechanisms against anti-aging are anti-inflammatory and antioxidant actions. Bacteroidetes, a phylum implicated in piglet diarrhea and colitis risk in mice [40], [41], was decreased in the Ca. In contrast, Firmicutes remained stable, consistent with their established role in producing SCFAs and modulating immune responses [33].

While phylum-level changes were subtle, genus-level analysis revealed more pronounced shifts in key microbial taxa (> 0.1 % relative abundances), suggesting targeted microbial community restructuring following probiotic supplementation. The Ca sows exhibited increased relative abundances of f_Clostridiaceae; g_*Clostridium*, g_SMB53, and g_*Treponema* and g_*Turicibacter*, while reduction in f_Ruminococcaceae_unclassified, f__Prevotellaceae;g_*Prevotella*, g_*Oscillospira*, f_S24–7_unclassified, o_Bacteroidales_unclassified, g_*Phascolarctobacterium*, and g_*Ruminococcus*, consisting with previously studies. These microbial alterations might relate to a shift in microbiota from the pro-inflammatory and opportunistic taxa to the possibly more health-promoting microbiota [4], [5], [40], [42]. The enrichment of *Clostridium*, family Clostridiaceae and *SMB53* (belongs family Clostridiaceae and Lachnospiraceae, respectively) is particularly relevant, as these genera are associated with butyrate production and intestinal epithelial health [33], [42]. Butyrate, a key SCFA, promotes mucosal barrier integrity, modulates inflammation, and serves as a primary energy source for colonocytes. Increased abundance of *Treponema* had been reported in healthy pigs and may contribute to fiber fermentation in the large intestine [43], and *Turicibacter* species could positively impact gut health by defending against enteropathogenic bacteria [40]. In contrast, *Prevotella*, a genus with pro-inflammatory properties, has been linked to piglet diarrhea [33]; and we found the lower of this group after the probiotic supplementation. High levels of *Prevotella* have been linked to increased production of succinate and mucin-degrading enzymes, which may disrupt gut barrier function and facilitate pathogen colonization [33], [44]. Consistently, the lower proportion of diarrhea in piglets suckled by these probiotic-supplemented sows compared with control-supplemented sows supported the Ca sows with modulated gut microbiota that affect piglet health outcome. The enhanced sows’ gut health and immune homeostasis might transfer to piglets via maternal immunity or microbial seeding at birth.

The NMDS analysis revealed distinct differences in gut microbiota compositions before and after probiotic supplementation at both genus and species levels [4], [45]. The microbial compositions were altered by key genera, including strong increase in f_Clostridiaceae;g_*Clostridium*, confirmed by LEfSe and species-level analysis, which further identified species as *C. butyricum* (the same species as the supplemented probiotic). Increases in other beneficial bacteria genera such as g_*Enterococcus* and g_*Lactobacillus* were also observed, consistent with their roles in mitigating piglet diarrhea [20], [33]. Other predominant bacteria in both Ca and Cb sows were similar, which might reflect the overall health status of the sows that were clinically healthy throughout the study.

Gut microbiota and potential microbial metabolic pathways allowed understand the impact of probiotic *C. butyricum* supplementation and its association with sow reproductive performance and piglet diarrhea. The comparative analysis of KEGG level 2 pathways revealed increased expression of amino acid metabolism, metabolism of other amino acids, carbohydrate metabolism, and eukaryotic cell communication. These functional changes corresponded well with the observed microbiota alterations in the Ca: for instances, many of the aforementioned genera in the Ca could ferment amino acids and complex carbohydrates into SCFAs, and these fermentation and metabolites support gut energy, gut epithelial barrier integrity, role in immune regulation and gut homeostasis [42], [46]. Conversely, pathways associated with infectious disease showed reduced expression after probiotic supplementation. These alterations suggest the potential functional contributions of gut microbiota in enhancing sow health, consistent with previous studies linking microbial metabolisms to improved animal health [47], [48], [49].

The relationship between alterations in sow gut microbial metabolism and reduced piglet diarrhea proportion prompted further examination of KEGG level 3 pathways. The Ca sows expressed increased expression of glycine, serine, and threonine metabolism, propanoate metabolism, and butanoate metabolism, alongside decreased expression of alanine, aspartate, and glutamate metabolism and galactose metabolisms. The SCFAs, such as acetate, propionate, and butyrate, are products during carbohydrate and amino acid fermentation by intestinal bacteria, especially lactic acid bacteria. These metabolites play an important role in metabolic homeostasis, gut integrity improvement, and overall gut health in humans and animals [47], [50]. Shin et al. [51] also reported the effects of probiotic supplementation (by *Lactobacillus* spp*.*) to increase population of lactic acid bacteria, and the butyrate and propanoate metabolisms predicted by PICRUSt analysis. The alanine, aspartate, and glutamate are amino acids involved in fatty acid synthesis and energy sources [52], and sometimes were reported decreased after probiotic supplementation [47]. Our study also found the increase for the eukaryote cellular process tight junction and adherens junction statistically higher in the Ca, supporting the improved sow gut health [49]. For instances, some SCFAs, e.g. butyrate is involved in various immune responses to prevent inflammation and oxidative stress in the colon [51]. Correlation analyses confirmed positive associations between butanoate metabolism, propanoate metabolism, tight junction expression (i.e., enhanced tight-junction assembly via AMP-activated protein kinase (AMPK) activation, enhanced junctional proteins like occluding and claudin) [53], [54], and the Ca microbiota. Interestingly, infectious diseases related to diarrhea, i.e., *S. aureus* infection [55], [56], were significantly lower after probiotic supplementation, meanwhile finding of *E. coli* could confer possibility for mutual or pathogenic bacteria depending on strains [1]. Overall, the findings suggest that maternal probiotic supplementation alters gut microbiota, promoting SCFAs (butanoate and propanoate) production and enhancing intestinal barrier function, which might contribute to reduce piglet diarrhea; consistent with previously report of the potential transfer of butanoate, propanoate, and beneficial bacteria from sows to their piglets by Chen et al. [57].

Sow health and reproductive performances are influenced by various factors, including probiotic supplement [58], a combination of *Bacillus mesentericus*, *C. butyricum*, and *Enterococcus faecalis* with peptide-zinc complexes has been shown to improve sows’ performance and prevent post-weaning diarrhea in piglets [59]. Our result found that feces score on day 10 (FEC_10) was inversely correlated with the Ca, gut microbiota after probiotic supplement [60]. Additionally, LSD3 demonstrated a positive correlation with the Ca microbiota, consistent with studies reporting an increase in piglet survival [61], [62], [63]. Maternal supplementation with probiotics influenced milk compositions, with higher total solid, casein, protein, and lactose contents being positively correlated with the Ca gut microbiota. The higher contents of protein and lactose after probiotic supplements were also found in previous studies [58], [64], [65]. Protein, the main constituent (∼15 %) of sow's milk, consists of whey and casein fractions that supply amino acids (AA) and bioactive factors essential for neonatal development [66]. Casein forms a clot in the stomach, slowing AA release to support digestion and absorption [66], [67]. Lactose, which accounts for 3–4 % of total milk, plays a crucial role in piglets’ growth since it is a palatable and easily digestible source of energy [67], [68], [69]. Previous studies reported that gestating sows supplemented probiotics before the expected farrowing date and during the lactation improved milk quality with higher fat and protein contents, thereby supporting the nutritional requirements of piglets and contributing to their improved health and performance [67], [70].

Investigating the relationship between changes in the milk metabolome and gut microbiota structure following probiotic supplementation in sows is key to identifying biomarkers linked to diarrhea reduction. In this study, the top 25 influential metabolites associated with gut microbial alterations were identified on day 17. Most of the metabolites found in sow milk differed from potential fecal metabolisms, as previously reported [57], possibly due to differences in microbiome composition. Considering that within the intestines, the sow diet contains more complex proteins and carbohydrates than those found in sow milk. Thus, the gut microbiome has a complex composition and differs from that found in milk [57]. While there are limited reports on the impact of milk metabolites on sow health [71], [72], our findings demonstrated that glutamate positively correlated with gut microbiota before probiotic supplementation. Elevated glutamate levels in piglets have been associated with piglet diarrhea, as well as a higher relative abundance of *Prevotella* in the gut [73].

These findings suggest that probiotic supplements in sows during late gestation and lactation alter gut microbiota, intestinal microbial functions, and milk metabolite composition, potentially reducing piglet diarrhea. Although the exact mechanism remains unclear, it may be due to (1) during gestation, the maternal gut microbiota influencing intrauterine microbiota colonization and providing beneficial metabolites that support fetal immune function and the development of piglet gut microbiota; (2) during farrowing, balanced gut microbiota or the presence of beneficial bacteria in the sow’s feces may reduce the exposure of piglets to pathogenic bacteria, facilitating rapid colonization of the piglet gut and promoting a healthy intestinal immune system; and (3) during lactation, the presence of beneficial metabolites in sow milk could enhance piglet health, potentially lowering the incidence of diarrhea. However, the study was limited by no fecal samplings immediately before and after farrowing, when stress and hormones might affect the gut microbiota dynamic and partly hindered the gut microbiota effect by *C. butyricum* supplementation [74]. Additionally, the study longitudinally analyzed gut microbiota from the same individual sows, so the correlations with sow reproductive performances and milk quality profiles were from differential from the probiotic-supplemented sows vs. the control-supplemented sows (from respective same breed and housing environment).

## 5 Conclusions

Maternal supplementation with *C. butyricum* significantly altered the gut microbial composition of sows, increasing beneficial bacteria such as *Clostridium, Bacillus, Lactobacillus,* and *Enterococcus*, while reducing potential pathogens like *Prevotella* and *Oscillospira*. These microbiota shifts were associated with enhanced microbial metabolisms, particularly in amino acid and short-chain fatty acid pathways, which contribute to gut health. Moreover, the supplementation also led to improvements in sow milk compositions, increasing protein, lactose, and total solids. Notably, piglets suckled by supplemented sows exhibited a lower proportion of diarrhea, particularly at day 17, suggesting a potential vertical transmission of beneficial microbes and metabolites from mother to offspring. The findings support the beneficial effects of *C. butyricum* as a probiotic supplement in sows, demonstrating its potential to enhance gut microbiota, improve milk quality, and reduce piglet diarrhea. This suggests its practical application as an alternative to antibiotics in improving swine health and productivity.

## CRediT authorship contribution statement

**Sissades Tongsima:** Supervision. **Sarn Settachaimongkon:** Methodology, Investigation. **Naraporn Somboonna:** Writing – review & editing, Writing – original draft, Visualization, Validation, Supervision, Resources, Project administration, Methodology, Investigation, Funding acquisition, Formal analysis, Data curation, Conceptualization. **Morakot Nuntapaitoon:** Writing – review & editing, Writing – original draft, Resources, Methodology, Investigation, Formal analysis, Data curation, Conceptualization. **Piraya Chatthanathon:** Visualization, Validation, Methodology, Investigation, Data curation. **Matanee Palasuk:** Methodology. **Alisa Wilantho:** Visualization, Data curation. **Jakavat Ruampatana:** Methodology, Investigation.

## CRediT authorship contribution statement

MN, PC, MP, AW, JR, SS and NS performed experiments and data analyses. MN designed the experimental study and wrote the manuscript. NS conceived and designed the study, coordinated the experiments and data analysis, and wrote the manuscript. ST advised. All authors read and approved the final manuscript.

## Declaration of Competing Interest

The authors declare that they have no known competing financial interests or personal relationships that could have appeared to influence the study reported in this paper. The authors have no conflict of interest.

## Data Availability

The 16S rRNA gene sequences in this study were deposited in the NCBI open access Sequence Read Archive database (accession number PRJNA1209561).

## Footnotes

Appendix A Supplementary data associated with this article can be found in the online version at doi:10.1016/j.csbj.2025.06.040.
