# Supplementary material for: Maternal Clostridium butyricum supplementation during late gestation and lactation enhances gut bacterial communities, milk quality, and reduces piglet diarrhea
Source: Comput Struct Biotechnol J. 2025 Jun 25;27:2933–45. doi: 10.1016/j.csbj.2025.06.040 (PMC12273217; doi:10.1016/j.csbj.2025.06.040)
Supplement: Supplementary file 1 — Supplementary material [file mmc1.docx]

**Supporting information**


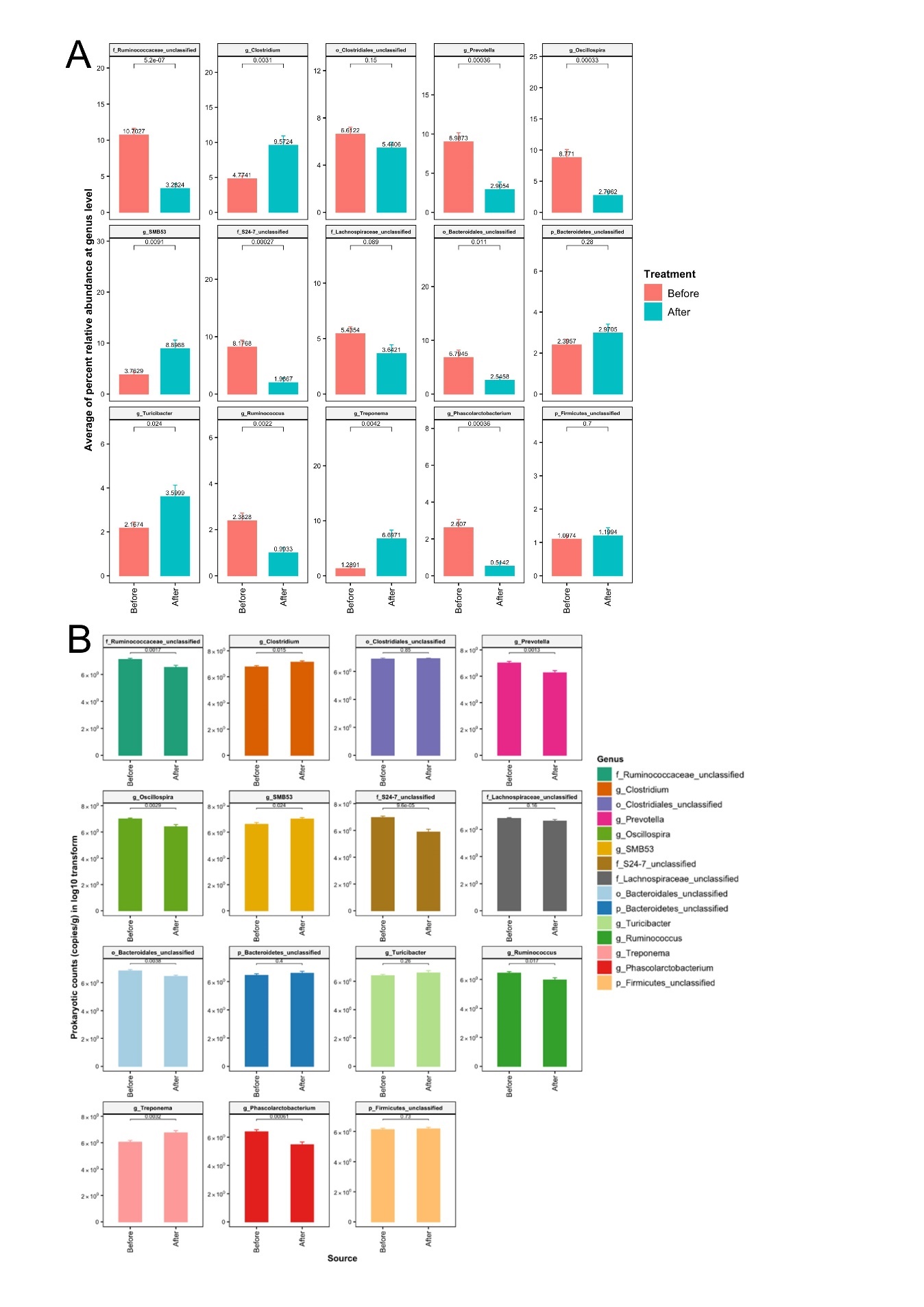


**Fig. S1.** Sows’ gut bacterial compositions in relative percentages (**A**) and copy numbers (**B**), at genus level. The 15 top dominant bacterial genus OTUs in relative percentages and copy numbers of the Cb vs. Ca groups, were compared for statistical difference by student’s *t*-test (*P* < 0.05). At genus level, OTUs were classified to the deepest taxonomic level where allowed. k_ abbreviates kingdom; p_, phylum; c_, class; o_, order; f_, family; and g_, genus.


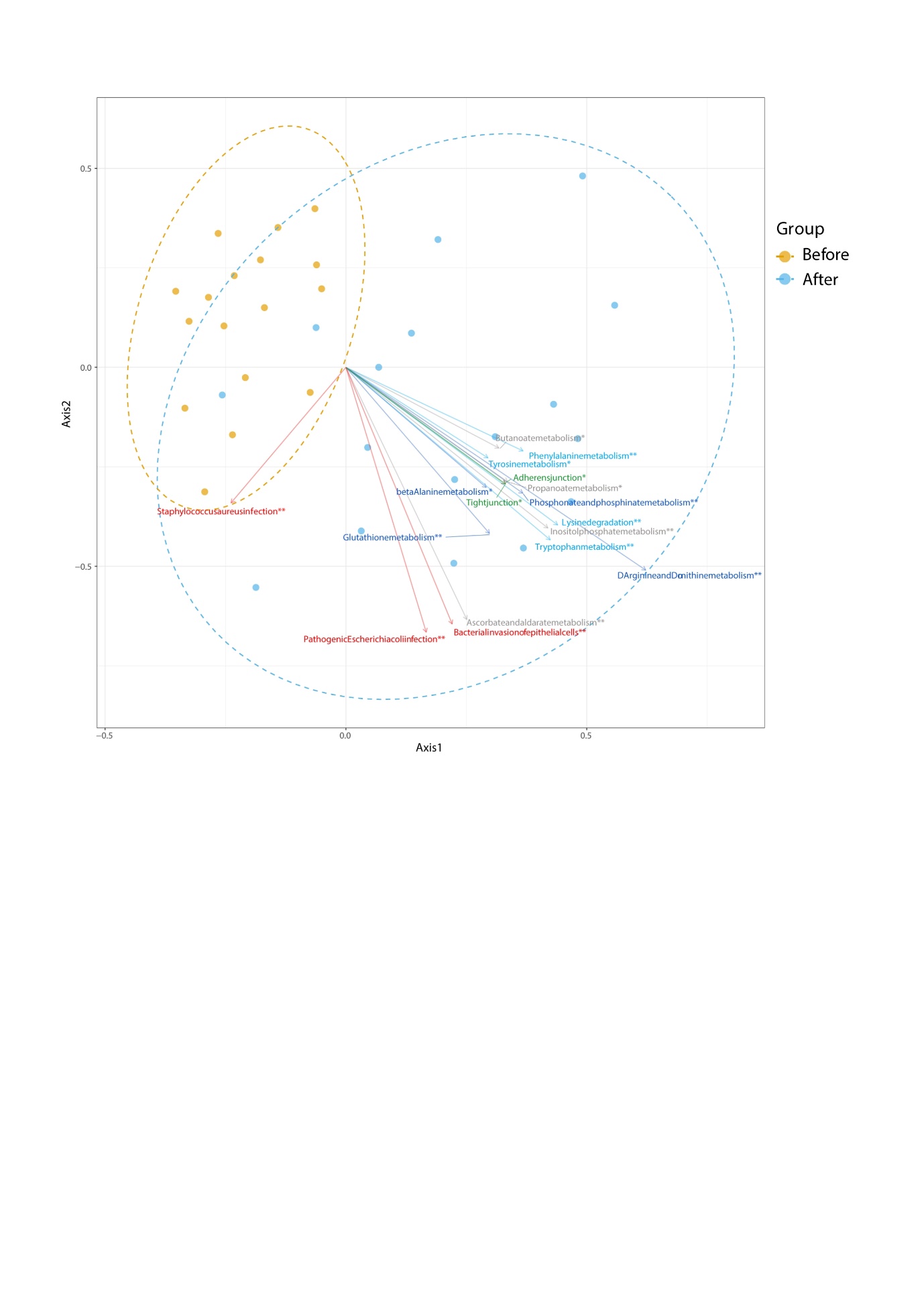


**Fig. S2.** Beta-diversity NMDS at species OTU level, along the Pearson's correlation with microbial metabolism potentials at KEGG pathway 3^rd^ level (Fig. 5B) (* indicates *P* ≤ 0.1, and ** indicates *P* ≤ 0.05). Arrows indicate the direction and magnitude of correlation of each factor to the Ca (or Cb) gut microbiota. Arrow color represents the KEGG pathway category: blue represents category amino acid metabolism; navy, metabolism of other amino acid; gray, carbohydrate metabolism; green, cell communication; and red, infectious disease. Noted that only metabolisms related to pig were displayed (i.e. no human disease displayed).

**Table S1.** Pearson's correlations of bacterial community structures with microbial metabolic potentials.

| level1 | level2 | level3 | axis1 | p-value | axis2 | p-value | length |
| --- | --- | --- | --- | --- | --- | --- | --- |
| Metabolism | Amino Acid Metabolism | Alanine aspartate and glutamate metabolism | -0.09039 | 0.603595 | 0.091611 | 0.598703 | 0.128696 |
|  |  | Amino acid related enzymes | -0.09677 | 0.578262 | 0.097539 | 0.575263 | 0.137401 |
|  |  | Arginine and proline metabolism | 0.140748 | 0.418782 | -0.03932 | 0.821308 | 0.146137 |
|  |  | Cysteine and methionine metabolism | 0.016975 | 0.922318 | 0.006882 | 0.968465 | 0.018317 |
|  |  | Glycine serine and threonine metabolism | 0.201591 | 0.246843 | -0.14041 | 0.4199 | 0.24567 |
|  |  | Histidine metabolism | -0.10889 | 0.531613 | 0.151258 | 0.384896 | 0.186378 |
|  |  | Lysine biosynthesis | -0.13226 | 0.447385 | 0.159081 | 0.360795 | 0.206881 |
|  |  | Lysine degradation | 0.439558 | 0.009293 | -0.39602 | 0.020424 | 0.591642 |
|  |  | Phenylalanine metabolism | 0.367605 | 0.032449 | -0.21002 | 0.227645 | 0.423367 |
|  |  | Phenylalanine tyrosine and tryptophan biosynthesis | -0.1445 | 0.406497 | 0.193939 | 0.265239 | 0.241851 |
|  |  | Tryptophan metabolism | 0.424168 | 0.012406 | -0.43353 | 0.010447 | 0.606522 |
|  |  | Tyrosine metabolism | 0.294845 | 0.090253 | -0.22757 | 0.191298 | 0.372453 |
|  |  | Valine leucine and isoleucine biosynthesis | -0.03132 | 0.857208 | 0.1178 | 0.498591 | 0.121893 |
|  |  | Cyanoamino acid metabolism | -0.13675 | 0.432135 | 0.071047 | 0.683177 | 0.154101 |
| Metabolism | Metabolism of Other Amino Acids | beta-Alanine metabolism | 0.291218 | 0.094343 | -0.30177 | 0.082837 | 0.419369 |
|  |  | Cyanoamino acid metabolism | -0.12629 | 0.468157 | 0.068649 | 0.693316 | 0.143742 |
|  |  | D-Alanine metabolism | -0.16858 | 0.332838 | 0.158942 | 0.361216 | 0.231692 |
|  |  | D-Arginine and D-ornithine metabolism | 0.622458 | 0.000085 | -0.50943 | 0.002089 | 0.804349 |
|  |  | D-Glutamine and D-glutamate metabolism | -0.14324 | 0.410595 | 0.138195 | 0.427272 | 0.199036 |
|  |  | Glutathione metabolism | 0.298028 | 0.086889 | -0.41724 | 0.014087 | 0.512748 |
|  |  | Phosphonate and phosphinate metabolism | 0.367185 | 0.032662 | -0.31622 | 0.068463 | 0.48458 |
|  |  | Selenocompound metabolism | 0.033005 | 0.849624 | 0.022462 | 0.897332 | 0.039923 |
|  |  | Taurine and hypotaurine metabolism | 0.039624 | 0.819941 | -0.0843 | 0.628213 | 0.093144 |
| Metabolism | Carbohydrate Metabolism | Amino sugar and nucleotide sugar metabolism | -0.19188 | 0.270342 | 0.059323 | 0.733267 | 0.200842 |
|  |  | Ascorbate and aldarate metabolism | 0.251038 | 0.149273 | -0.63351 | 0.000058 | 0.681439 |
|  |  | Butanoate metabolism | 0.317456 | 0.067328 | -0.20277 | 0.244095 | 0.376687 |
|  |  | C5-Branched dibasic acid metabolism | -0.02699 | 0.876804 | 0.159461 | 0.359648 | 0.161729 |
|  |  | Citrate cycle (TCA cycle) | 0.126205 | 0.468457 | -0.16644 | 0.339017 | 0.208876 |
|  |  | Fructose and mannose metabolism | -0.19485 | 0.26301 | -0.05827 | 0.737823 | 0.203373 |
|  |  | Galactose metabolism | -0.18565 | 0.286457 | -0.08234 | 0.636553 | 0.203085 |
|  |  | Glycolysis / Gluconeogenesis | 0.066065 | 0.704305 | -0.13259 | 0.446259 | 0.148137 |
|  |  | Glyoxylate and dicarboxylate metabolism | 0.279351 | 0.108479 | -0.22409 | 0.198171 | 0.358127 |
|  |  | Inositol phosphate metabolism | 0.419182 | 0.0136 | -0.40374 | 0.017889 | 0.581994 |
|  |  | Pentose and glucuronate interconversions | 0.198791 | 0.253468 | -0.15576 | 0.370924 | 0.252542 |
|  |  | Pentose phosphate pathway | 0.041154 | 0.813115 | -0.00015 | 0.999299 | 0.041154 |
|  |  | Propanoate metabolism | 0.335529 | 0.052388 | -0.2876 | 0.098511 | 0.441918 |
|  |  | Pyruvate metabolism | 0.16733 | 0.33625 | -0.12236 | 0.482417 | 0.207296 |
|  |  | Starch and sucrose metabolism | -0.13692 | 0.431536 | 0.076341 | 0.660992 | 0.156767 |
| Cellular Processes | Cell Communication | Adherens junction | 0.331271 | 0.055618 | -0.29305 | 0.092651 | 0.442286 |
|  |  | Focal adhesion | 0.249711 | 0.149037 | -0.24971 | 0.157229 | 0.353145 |
|  |  | Tight junction | 0.331271 | 0.055618 | -0.29305 | 0.092651 | 0.442286 |
| Human Diseases | Infectious Diseases | African trypanosomiasis | -0.38246 | 0.025594 | 0.436397 | 0.009873 | 0.580273 |
|  |  | Amoebiasis | 0.57682 | 0.000356 | -0.20002 | 0.250556 | 0.610514 |
|  |  | Bacterial invasion of epithelial cells | 0.220507 | 0.205256 | -0.64471 | 0.000039 | 0.681379 |
|  |  | Chagas disease | -0.06713 | 0.699767 | -0.14757 | 0.396608 | 0.162117 |
|  |  | Epithelial cell signaling in Helicobacter pylori infection | -0.24034 | 0.167394 | 0.22139 | 0.203447 | 0.326765 |
|  |  | InfluenzaA | 0.669601 | 0.000015 | -0.5609 | 0.000559 | 0.873484 |
|  |  | Pathogenic Escherichia coli infection | 0.174425 | 0.316346 | -0.65887 | 0.000023 | 0.681567 |
|  |  | Pertussis | -0.25042 | 0.150277 | -0.27364 | 0.115959 | 0.370933 |
|  |  | Shigellosis | 0.166679 | 0.338315 | -0.66534 | 0.000018 | 0.685902 |
|  |  | Staphylococcus aureus infection | -0.23852 | 0.170625 | -0.34059 | 0.048716 | 0.415806 |
|  |  | Toxoplasmosis | 0.508536 | 0.002134 | -0.24183 | 0.164775 | 0.563107 |
|  |  | Tuberculosis | -0.03606 | 0.83589 | 0.084346 | 0.628011 | 0.091731 |
|  |  | Vibrio cholerae infection | -0.00419 | 0.980816 | 0.096434 | 0.579599 | 0.096525 |
|  |  | Vibrio cholerae pathogenic cycle | -0.10374 | 0.551202 | -0.05271 | 0.762037 | 0.116367 |

**Table S2.** Pearson's correlations of bacterial community structures with pig performances and milk components.

| Feature | Name | abbreviation | axis1 | p-value | axis2 | p-value | length |
| --- | --- | --- | --- | --- | --- | --- | --- |
| Clinical data | prenatal spinal fat | BF1 | 0.006193 | 0.972053 | -0.091891 | 0.6032 | 0.092099 |
|  | spinal fat after weaning | BF2 | -0.001843 | 0.991683 | -0.20588 | 0.24417 | 0.205889 |
|  | total number of piglets at birth | TB | 0.090416 | 0.609024 | 0.083993 | 0.63469 | 0.123409 |
|  | number of live newborn piglets | BA | 0.24088 | 0.173002 | -0.015812 | 0.92873 | 0.241398 |
|  | number of dead newborn piglets | SB | 0.079138 | 0.654391 | 0.081449 | 0.64498 | 0.113564 |
|  | mummy | MM | -0.190802 | 0.280436 | -0.056187 | 0.75061 | 0.198903 |
|  | litter size on 24-hour | LS24h | 0.304632 | 0.084754 | -0.10816 | 0.54064 | 0.323264 |
|  | litter size on day 3 | LSD3 | 0.376761 | 0.030678 | -0.151985 | 0.38992 | 0.406261 |
|  | litter weight on day 3 | LWD3 | 0.237152 | 0.179747 | -0.034261 | 0.84633 | 0.239614 |
|  | average litter weight on day 3-10 | LWG_310 | -0.167826 | 0.420897 | -0.012174 | 0.95344 | 0.168267 |
|  | litter size on day 10 | LSD10 | 0.096405 | 0.585216 | 0.035394 | 0.84095 | 0.102697 |
|  | litter weight on day 10 | LWD10 | 0.189171 | 0.284568 | 0.104947 | 0.55274 | 0.216332 |
|  | average litter weight on day 10-17 | LWG_1017 | 0.219231 | 0.28282 | 0.26 | 0.20276 | 0.340091 |
|  | litter size on day 17 | LSD17 | 0.157682 | 0.372403 | 0.11763 | 0.50578 | 0.196724 |
|  | litter weight on day 17 | LWD17 | 0.251003 | 0.155641 | 0.17246 | 0.32927 | 0.30454 |
|  | litter size on day 21 | LSD21 | 0.21271 | 0.228871 | 0.114938 | 0.51557 | 0.241777 |
|  | litter weight on day 21 | LWD21 | 0.290775 | 0.099996 | 0.125 | 0.4795 | 0.316505 |
|  | number of weaned pigs | WP | 0.238991 | 0.176396 | 0.085113 | 0.63018 | 0.253694 |
|  | weaned pig weight | WWT | 0.268954 | 0.128151 | 0.123682 | 0.48415 | 0.29603 |
|  | milk yield on day 3-10 | MY3_10 | -0.073043 | 0.72611 | -0.14 | 0.50196 | 0.157909 |
|  | milk yield on day 10-17 | MY10_17 | 0.33258 | 0.12749 | 0.188029 | 0.38888 | 0.382053 |
|  | feces score on day 10 | FEC_10 | -0.594398 | 0.002479 | 0.504919 | 0.00163 | 0.779905 |
|  | the amount of IgG2 | IgG2 | 0.277922 | 0.213903 | 0.241558 | 0.28002 | 0.368227 |
|  | the amount of IgA | IgA | 0.233766 | 0.295822 | 0.098701 | 0.65892 | 0.253749 |
| Milk components | fat on day 0 | fat_d0 | 0.248641 | 0.243522 | 0.133959 | 0.52979 | 0.282431 |
|  | fat on day 3 | fat_d3 | -0.032572 | 0.865603 | 0.181744 | 0.34498 | 0.184639 |
|  | fat on day 17 | fat_d17 | -0.159278 | 0.40788 | 0.219485 | 0.25409 | 0.271189 |
|  | protein on day 0 | protein_d0 | 0.645257 | 0.000885 | -0.159091 | 0.45555 | 0.66458 |
|  | protein on day 3 | protein_d3 | 0.078818 | 0.682137 | 0.252326 | 0.18982 | 0.26435 |
|  | protein on day 17 | protein_d17 | 0.010947 | 0.954639 | 0.249042 | 0.19564 | 0.249283 |
|  | casein on day 0 | casein_d0 | 0.63405 | 0.001159 | -0.164566 | 0.44018 | 0.655058 |
|  | casein on day 3 | casein_d3 | 0.120175 | 0.532333 | 0.220367 | 0.25218 | 0.251005 |
|  | casein on day 17 | casein_d17 | 0.139045 | 0.469989 | 0.101546 | 0.59774 | 0.172178 |
|  | lactose on day 0 | lactose_d0 | -0.466634 | 0.024792 | -0.057341 | 0.78797 | 0.470144 |
|  | lactose on day 3 | lactose_d3 | 0.367866 | 0.054108 | -0.17928 | 0.35156 | 0.409227 |
|  | lactose on day 17 | lactose_d17 | 0.412151 | 0.029303 | -0.063492 | 0.74147 | 0.417013 |
|  | total solid on day 0 | TS_d0 | 0.522727 | 0.010494 | 0.124506 | 0.55923 | 0.53735 |
|  | total solid on day 3 | TS_d3 | 0.003011 | 0.987518 | 0.217052 | 0.25939 | 0.217073 |
|  | total solid on day 17 | TS_d17 | -0.095525 | 0.619639 | 0.182291 | 0.34353 | 0.205803 |

**Table S3.** Pearson's correlations of bacterial community structures with milk metabolome profiles.

| Day | Feature | axis1 | p-value | axis2 | p-value | length |
| --- | --- | --- | --- | --- | --- | --- |
| Day 0 | Acetate_d0 | -0.281818 | 0.372829 | 0.154545 | 0.625043 | 0.321412 |
|  | Adenine_d0 | -0.309091 | 0.328356 | 0.227273 | 0.472326 | 0.383654 |
|  | Alanine_d0 | -0.218182 | 0.490225 | 0.209091 | 0.508482 | 0.302196 |
|  | Betain_d0 | -0.145455 | 0.645539 | 0.127273 | 0.687337 | 0.193275 |
|  | Biotin_d0 | -0.045455 | 0.885706 | 0.054545 | 0.863054 | 0.071002 |
|  | Carnitine_d0 | -0.354545 | 0.262215 | -0.036364 | 0.908451 | 0.356405 |
|  | Choline_d0 | -0.272727 | 0.388446 | 0.027273 | 0.931272 | 0.274088 |
|  | Citrate_d0 | -0.263636 | 0.404455 | 0.218182 | 0.490225 | 0.34221 |
|  | Creatine_d0 | -0.172727 | 0.58492 | 0.127273 | 0.687337 | 0.214553 |
|  | Creatinephosphate_d0 | -0.172727 | 0.58492 | 0.072727 | 0.818104 | 0.187414 |
|  | Creatinine_d0 | -0.327273 | 0.300703 | 0.181818 | 0.565319 | 0.374387 |
|  | Dimethylamine_d0 | -0.290909 | 0.357607 | 0.145455 | 0.645539 | 0.325246 |
|  | Glutamate_d0 | -0.127273 | 0.687337 | 0.145455 | 0.645539 | 0.193275 |
|  | Glycine_d0 | -0.290909 | 0.357607 | 0.090909 | 0.773745 | 0.304783 |
|  | Glycolate_d0 | -0.172727 | 0.58492 | 0.072727 | 0.818104 | 0.187414 |
|  | Hypoxanthine_d0 | -0.309091 | 0.328356 | 0.227273 | 0.472326 | 0.383654 |
|  | Lactate_d0 | -0.163636 | 0.604833 | -0.027273 | 0.931272 | 0.165894 |
|  | Lactose_d0 | -0.181818 | 0.565319 | 0.109091 | 0.730113 | 0.212035 |
|  | myo_Inositol_d0 | -0.272727 | 0.388446 | 0.136364 | 0.666309 | 0.304918 |
|  | methanol_d0 | -0.372727 | 0.238531 | 0.345455 | 0.274647 | 0.508197 |
|  | N_Acetylglucosamine_d0 | -0.309091 | 0.328356 | 0.172727 | 0.58492 | 0.354079 |
|  | N_Acetylglutamate_d0 | -0.272727 | 0.388446 | 0.227273 | 0.472326 | 0.355011 |
|  | O_Acetylcarnitine_d0 | 0.009091 | 0.977066 | 0.118182 | 0.70861 | 0.118531 |
|  | O_Acteylcholine_d0 | -0.327273 | 0.300703 | 0.045455 | 0.885706 | 0.330414 |
|  | O_phosphocholine_d0 | -0.272727 | 0.388446 | 0.027273 | 0.931272 | 0.274088 |
|  | Ribose_d0 | -0.272727 | 0.388446 | 0.145455 | 0.645539 | 0.309091 |
|  | Threonine_d0 | -0.109091 | 0.730113 | -0.154545 | 0.625043 | 0.18917 |
|  | sn_Glycero_3_phosphocoline_d0 | -0.272727 | 0.388446 | 0.027273 | 0.931272 | 0.274088 |
|  | taurine_d0 | 0 | 1 | 0.272727 | 0.388446 | 0.272727 |
|  | UDP_Galactose_d0 | -0.236364 | 0.454794 | 0.181818 | 0.565319 | 0.298204 |
|  | UDP_Glucose_d0 | -0.172727 | 0.58492 | 0.118182 | 0.70861 | 0.209288 |
|  | UDP_N_Acetylglucosamine_d0 | -0.245455 | 0.437634 | 0.045455 | 0.885706 | 0.249628 |
|  | UMP_d0 | -0.209091 | 0.508482 | 0.090909 | 0.773745 | 0.227999 |
|  | Uracil_d0 | -0.318182 | 0.314329 | 0.109091 | 0.730113 | 0.336364 |
|  | Uridine_d0 | -0.1 | 0.75183 | 0.172727 | 0.58492 | 0.199586 |
| Day 3 | Acetate_d3 | -0.327273 | 0.300703 | -0.190909 | 0.546038 | 0.378885 |
|  | Adenine_d3 | -0.263636 | 0.404455 | 0.054545 | 0.863054 | 0.26922 |
|  | Alanine_d3 | -0.254545 | 0.420852 | -0.1 | 0.75183 | 0.273484 |
|  | Betain_d3 | 0.281818 | 0.372829 | -0.081818 | 0.795842 | 0.293455 |
|  | Biotin_d3 | -0.309091 | 0.328356 | 0.063636 | 0.840514 | 0.315574 |
|  | Carnitine_d3 | 0.036364 | 0.908451 | -0.1 | 0.75183 | 0.106406 |
|  | Choline_d3 | 0.036364 | 0.908451 | -0.027273 | 0.931272 | 0.045455 |
|  | Citrate_d3 | -0.018182 | 0.95415 | -0.054545 | 0.863054 | 0.057496 |
|  | Creatine_d3 | 0.063636 | 0.840514 | -0.118182 | 0.70861 | 0.134226 |
|  | Creatinephosphate_d3 | 0.272727 | 0.388446 | -0.109091 | 0.730113 | 0.293736 |
|  | Creatinine_d3 | -0.3 | 0.342782 | -0.145455 | 0.645539 | 0.333402 |
|  | Dimethylamine_d3 | -0.018182 | 0.95415 | 0 | 1 | 0.018182 |
|  | Glutamate_d3 | 0.290909 | 0.357607 | -0.118182 | 0.70861 | 0.313998 |
|  | Glycine_d3 | 0.327273 | 0.300703 | 0 | 1 | 0.327273 |
|  | Glycolate_d3 | 0.272727 | 0.388446 | -0.109091 | 0.730113 | 0.293736 |
|  | Hypoxanthine_d3 | -0.263636 | 0.404455 | 0.054545 | 0.863054 | 0.26922 |
|  | Lactate_d3 | -0.427273 | 0.176646 | -0.136364 | 0.666309 | 0.448505 |
|  | Lactose_d3 | 0.290909 | 0.357607 | -0.118182 | 0.70861 | 0.313998 |
|  | myo-Inositol_d3 | 0.072727 | 0.818104 | -0.181818 | 0.565319 | 0.195824 |
|  | methanol_d3 | -0.318182 | 0.314329 | -0.081818 | 0.795842 | 0.328533 |
|  | N-Acetylglucosamine_d3 | -0.2 | 0.527089 | -0.090909 | 0.773745 | 0.219692 |
|  | N-Acetylglutamate_d3 | -0.245455 | 0.437634 | -0.109091 | 0.730113 | 0.268605 |
|  | O-Acetylcarnitine_d3 | -0.254545 | 0.420852 | -0.236364 | 0.454794 | 0.347363 |
|  | O-Acteylcholine_d3 | -0.109091 | 0.730113 | -0.163636 | 0.604833 | 0.196666 |
|  | O-phosphocholine_d3 | 0.036364 | 0.908451 | -0.027273 | 0.931272 | 0.045455 |
|  | Ribose_d3 | -0.536364 | 0.088953 | 0.018182 | 0.95415 | 0.536672 |
|  | Threonine_d3 | -0.318182 | 0.314329 | -0.127273 | 0.687337 | 0.342692 |
|  | sn-Glycero-3-phosphocoline_d3 | -0.172727 | 0.58492 | -0.081818 | 0.795842 | 0.191125 |
|  | taurine_d3 | -0.154545 | 0.625043 | 0.036364 | 0.908451 | 0.158766 |
|  | UDP-Galactose_d3 | -0.236364 | 0.454794 | 0.136364 | 0.666309 | 0.272879 |
|  | UDP-Glucose_d3 | -0.263636 | 0.404455 | -0.036364 | 0.908451 | 0.266132 |
|  | UDP-N-Acetylglucosamine_d3 | -0.227273 | 0.472326 | -0.090909 | 0.773745 | 0.24478 |
|  | UMP_d3 | 0 | 1 | -0.045455 | 0.885706 | 0.045455 |
|  | Uracil_d3 | -0.4 | 0.205903 | -0.163636 | 0.604833 | 0.432177 |
|  | Uridine_d3 | 0.1 | 0.75183 | -0.127273 | 0.687337 | 0.161859 |
| Day 17 | Acetate_d17 | -0.663636 | 0.025984 | 0.181818 | 0.565319 | 0.688092 |
|  | Adenine_d17 | -0.618182 | 0.042646 | 0.272727 | 0.388446 | 0.675669 |
|  | Alanine_d17 | -0.554545 | 0.076652 | 0.172727 | 0.58492 | 0.580823 |
|  | Betain_d17 | -0.7 | 0.016471 | 0.327273 | 0.300703 | 0.772727 |
|  | Biotin_d17 | -0.490909 | 0.120569 | -0.136364 | 0.666309 | 0.509497 |
|  | Carnitine_d17 | -0.745455 | 0.008455 | -0.027273 | 0.931272 | 0.745953 |
|  | Choline_d17 | -0.763636 | 0.006233 | -0.072727 | 0.818104 | 0.767092 |
|  | Citrate_d17 | -0.518182 | 0.101289 | 0.009091 | 0.977066 | 0.518262 |
|  | Creatine_d17 | -0.727273 | 0.011205 | 0.309091 | 0.328356 | 0.79023 |
|  | Creatinephosphate_d17 | -0.636364 | 0.035287 | 0.336364 | 0.287476 | 0.719791 |
|  | Creatinine_d17 | -0.690909 | 0.018565 | 0.172727 | 0.58492 | 0.712173 |
|  | Dimethylamine_d17 | -0.609091 | 0.046696 | -0.072727 | 0.818104 | 0.613417 |
|  | Glutamate_d17 | -0.6 | 0.051003 | 0.381818 | 0.227272 | 0.711186 |
|  | Glycine_d17 | -0.636364 | 0.035287 | 0.336364 | 0.287476 | 0.719791 |
|  | Glycolate_d17 | -0.7 | 0.016471 | 0.3 | 0.342782 | 0.761577 |
|  | Hypoxanthine_d17 | -0.618182 | 0.042646 | 0.272727 | 0.388446 | 0.675669 |
|  | Lactate_d17 | -0.318182 | 0.314329 | 0.109091 | 0.730113 | 0.336364 |
|  | Lactose_d17 | -0.636364 | 0.035287 | 0.336364 | 0.287476 | 0.719791 |
|  | myo-Inositol_d17 | -0.672727 | 0.023313 | 0.281818 | 0.372829 | 0.729372 |
|  | methanol_d17 | -0.254545 | 0.420852 | -0.318182 | 0.314329 | 0.407472 |
|  | N-Acetylglucosamine_d17 | -0.790909 | 0.003746 | 0.081818 | 0.795842 | 0.79513 |
|  | N-Acetylglutamate_d17 | -0.709091 | 0.014552 | 0.227273 | 0.472326 | 0.744623 |
|  | O-Acetylcarnitine_d17 | -0.572727 | 0.065543 | 0.054545 | 0.863054 | 0.575319 |
|  | O-Acteylcholine_d17 | -0.809091 | 0.002559 | -0.036364 | 0.908451 | 0.809908 |
|  | O-phosphocholine_d17 | -0.763636 | 0.006233 | -0.072727 | 0.818104 | 0.767092 |
|  | Ribose_d17 | -0.527273 | 0.095438 | 0.572727 | 0.065543 | 0.778481 |
|  | Threonine_d17 | -0.2 | 0.527089 | 0.045455 | 0.885706 | 0.2051 |
|  | sn-Glycero-3-phosphocoline_d17 | -0.790909 | 0.003746 | 0.045455 | 0.885706 | 0.792214 |
|  | taurine_d17 | -0.281818 | 0.372829 | -0.418182 | 0.186033 | 0.504279 |
|  | UDP-Galactose_d17 | -0.754545 | 0.007282 | 0.281818 | 0.372829 | 0.805457 |
|  | UDP-Glucose_d17 | -0.736364 | 0.00976 | 0.372727 | 0.238531 | 0.825322 |
|  | UDP-N-Acetylglucosamine_d17 | -0.727273 | 0.011205 | 0.354545 | 0.262215 | 0.809091 |
|  | UMP_d17 | -0.7 | 0.016471 | 0.254545 | 0.420852 | 0.744845 |
|  | Uracil_d17 | -0.845455 | 0.001045 | 0.463636 | 0.142608 | 0.964237 |
|  | Uridine_d17 | -0.636364 | 0.035287 | 0.336364 | 0.287476 | 0.719791 |
